# miR663 Prevents Epo Inhibition Caused by TNF-Alpha in Normoxia and Hypoxia

**DOI:** 10.1155/2021/3670499

**Published:** 2021-07-27

**Authors:** Mete Ozkurt, Thomas Hellwig-Bürgel, Reinhard Depping, Selda Kadabere, Rumeysa Ozyurt, Abdullah Karadag, Nilüfer Erkasap

**Affiliations:** ^1^Department of Physiology, Eskisehir Osmangazi University Medical Faculty, Eskisehir, Turkey; ^2^Institute of Physiology, University of Lubeck, Lubeck, Germany; ^3^Department of Physiology, Adiyaman University Medical Faculty, Adiyaman, Turkey

## Abstract

**Objective:**

In chronic inflammatory diseases, proinflammatory cytokines such as TNF-*α* are present in high amounts in the circulation and are associated with anemia in most cases. Experimental studies have shown that TNF-*α* inhibits the synthesis of erythropoietin (Epo), the main stimulant of hematopoiesis. Our aim was to figure out which microRNAs are involved in the Epo repression by TNF-*α*.

**Methods:**

First, we determined the dose of TNF-*α* in HepG2 cells that has no cytotoxic effect by using MTT assays and that inhibits Epo synthesis by qRT-PCR and ELISA. Then, we performed the microRNA array study with TNF-*α* (20 ng/ml)-treated cells, and the array results were confirmed by qRT-PCR. We transfected the miR663 group with the mimic-miR663 (30 pmol) for 24 hrs; other groups were treated with a transfection reagent followed by treatment of TNF-*α* for 24 hrs; miR663 groups were treated with TNF-*α* for 24 hrs; and the control group was incubated with normal medium. We analyzed Epo mRNA levels by qRT-PCR. If mimic-miR663 prevents the Epo repression by TNF-*α*, more Epo-dependent UT-7 cells would survive. Therefore, we cocultured HepG2 cells with UT-7 cells. The percentage of apoptotic UT-7 cells was determined by TUNEL assays.

**Results:**

According to our array study, TNF-*α* significantly decreases miR663 expression. After transfection of miR663 mimics into HepG2 cells, TNF-alpha was unable to decrease Epo mRNA amounts. Furthermore, mimic-miR663 transfection resulted in a lower apoptosis rate of UT-7 cells in coculture experiments.

**Conclusions:**

miR663 is involved in Epo mRNA production and that is able to prevent or reverse the inhibitory effect of TNF-*α*. In our coculture study, transfecting HepG2 cells with miR663 mimics decreased the apoptosis of UT-7 cells.

## 1. Introduction

In patients suffering from chronic inflammatory diseases such as rheumatoid arthritis (RA), proinflammatory cytokines such as TNF-alpha (TNF-*α*) are present in high concentrations in the circulation. These high proinflammatory cytokine levels often lead to the development of anemia (anemia of chronic disease, ACD) [1]. Song et al. stated that anemia was found in 66% of RA patients and adversely affected the symptoms of the disease in a clinical study [2].

Epo, which is synthesized by the liver in the fetal period and by renal peritubular fibroblasts after birth, is transmitted to the bone marrow through the blood circulation and stimulates erythropoiesis by enabling the differentiation of multipotent blood stem cells into erythrocytes via EpoR. In addition, Epo protects erythrocytes against apoptosis [3]. Since recombinant human erythropoietin (rHuEpo) treatment increases red blood cell counts in RA patients, rHuEpo treatment has become the clinical standard treatment for ACD [4]. However, rHuEpo treatment has been associated with high risk of cancer or cardiovascular disease [5–7].

TNF-*α* is a cytokine that plays a dominant role in the inflammatory response and is synthesized by many cell types but mostly macrophages [8]. Studies with the isolated perfused rat kidney model and human HepG2 cells have shown that TNF-*α* suppresses Epo synthesis in a dose-dependent manner [9]. TNF-*α* suppresses Epo expression by binding to the promoter region of Epo via GATA-2 and NfKappaB [10]. In vivo and in vitro studies on astrocytes have shown that TNF-*α* suppresses hypoxia-induced Epo expression also in a dose-dependent manner [11]. In our previous study, we have shown that elevated levels of TNF-*α* in hypertension decreased the amount of Epo in the circulation and that circulating Epo concentrations increased to normal values when TNF-*α* was blocked with anti-TNF-*α* antibodies [12].

miRNAs are small, noncoding, linear RNAs and have important roles in posttranscriptional gene regulation [13]. Because of their ability to influence mRNA stability, they generally have a significant effect on the amount of protein synthesis. Therefore, miRNAs have been an important area of interest since their discovery in 1993, so that the physiological and pathophysiological mechanisms can be deeply understood [14].

Although Epo has been extensively studied over the years, there are only a few studies on miRNAs involved in Epo metabolism yet. In a recent work by Rivkin et al., miRNA122 was shown to play a role in the inhibitory effect of TNF-*α* on Epo synthesis [15]. Additionally, Ferracin et al. demonstrated the inhibitory effect of miRNA125b on Epo expression in breast cancer [16]. Considering the complex mechanisms of protein synthesis and the thousands of miRNAs discovered, we can easily say that we are at the very beginning of understanding miRNA signaling.

We aimed to detect miRNAs involved in the inhibition of TNF-*α* on Epo synthesis. Hence, we first determined the optimal dose of TNF-*α* that inhibited Epo at both mRNA and protein levels in HepG2 cells. Next, we performed an array analysis with the TNF-*α* dose and determined and reviewed the result of the array over the data banks. Then, we did a confirmation study with miRNAs that were able to affect Epo mRNA levels; and after that, we transfected cells with the mimics of significantly downregulated miRNAs. Finally, we made a coculture study with Epo-producing HepG2 cells and Epo-dependent UT-7 cells.

## 2. Materials and Methods

### 2.1. Detection of Optimum Dose of TNF-*α* to Inhibit Epo Synthesis in HepG2 Cells

The human hepatocellular carcinoma cancer cell line HepG2 (the American Type Culture Collection (ATTC), Rockville, MD) was cultured in the Roswell Park Memorial Institute medium (RPMI, Sigma; St. Louis, MO) containing 10% fetal bovine serum (FBS; Sigma, St. Louis, MO), 100 U/ml penicillin (Sigma), and 100 *μ*g/ml streptomycin (Sigma).

Cell proliferation was detected by using the MTT assay with HepG2 cells seeded in 96-well plates, and 24 hours later, the cells were treated with TNF-*α* (1, 5, 10, 15, 20, 25, 30, 40, 50, 75, 100 ng/ml) or without TNF-*α* as a control for 24 hrs (*n* = 8). After 24 hours, the MTT solution was added into the medium (1/10 amount) and the cells were incubated for 4 hours at 37°C. After incubation, DMSO was added (100 *μ*l) and was shaken for 5 min. The number of living cells in the culture was determined by absorbance at 550 nm using a microplate reader (AMR-100; ALLSHENG, Hangzhou, China).

After detecting the safe dose as 10–30 ng/ml of TNF-*α* for HepG2 cells, we incubated the cells with 4 different doses of TNF-*α* (10, 15, 20, and 30 ng/ml) in 24-well plates. After 24 hours of incubation, we collected the cells and the cell culture media. Total RNA was isolated from the cells using the GeneJET RNA Purification Kit (Thermo Scientific, USA). The concentration and purity of the RNA were spectrometrically measured using NanoDrop 1000 (Thermo Scientific, USA). Isolated RNA samples were converted to complementary DNAs (cDNAs) using the RevertAid First Strand cDNA Synthesis Kit (Thermo Scientific, USA) at 42°C for 60 min and 70°C for 5 min. cDNA samples were stored at −80°C until further analysis. Epo expressions were measured using the TaqMan qRT-PCR Kit (Thermo Scientific, USA). cDNA synthesis was verified by the detection of the *β*-actin transcript, which was used as an internal control. Relative differences in expression were determined using the comparative threshold cycle (2^−ΔΔCt^) method. Epo proteins in the cell media were detected by using a commercially available ELISA kit (Cloud-Clone Corp., USA).

### 2.2. Detection and Confirmation of miRNA Profile Affecting Epo by Array Analysis

Isolated RNAs of all groups were run in agarose gels to confirm the quantity and quality (*n* = 6) of the samples. In the range of 0.1*–*3 *μ*g of total RNA samples were used for array studies. After adding poly-A tail to the miRNAs by using adequate enzymes and tampons diluted in 1 mM Tris solution, we did the array analysis using GeneChip miRNA 4.0 Array (Affymetrix, USA).

Through the report of the array study, those miRNAs which showed at least twofold change were analyzed using the databases mirbase.org and targetscan.org according to their potential to affect Epo synthesis. Our analysis demonstrated that seven miRNAs were downregulated (miR4459, miR663a, miR1908-5p, miR4433-3p, miR149-3p, miR4739, and miR6805-5p) and one miRNA (miR122-5p) was upregulated. Three out of seven downregulated miRNAs and one upregulated miRNA were taken for the confirmation study. qRT-PCR was performed by using the mirVana™ qRT-PCR miRNA Detection Kit (Thermo Scientific, USA). Relative differences in expression were determined using the comparative threshold cycle (2^−ΔΔCt^) method.

### 2.3. Incubation of HepG2 Cells with Mimic-miR663 in Hypoxia and Normoxia

To detect the optimum dose of mimic-miR663, after 24 hours of seeding the HepG2 cells on 24-well plates, the control and TNF-*α* groups were given a medium without antibody and 3 ml of transfection reagent (Lipofectamine™ RNAiMAX; Thermo Scientific, USA), while the miR663 group was treated with different doses (5, 15, 30, 60 pmol) of mimic-miR663 (Ambion, Thermo Scientific, USA), 3 ml of transfection agent, and a medium without antibody (*n* = 6). The transfection agent and the mimic-miR663 were first dissolved in a special medium (Opti-Mem). After 24 hours of transfection, TNF-*α* and miR663 groups were treated with 20 ng/ml of TNF-*α*, while the control group was treated with normal medium for 24 hours. We detected the optimum dose as 5 pmol and repeated the experiment with this dose. To induce hypoxia, the cells were incubated under 5% O_2_ for the last 6 hours of 24 hours.

At the end of the experiments, the cells were collected and total RNA isolated by using the innuPREP RNA mini kit (AnalytikJena, Germany), cDNAs were synthesized and qRT-PCR were performed the same way as in Section 2.1.

### 2.4. Coculture of HepG2 Cells and UT-7/Epo Cells and TUNEL Analysis of UT-7/Epo Cells

After 6 hours of incubation of the cells with TNF-*α* by following the same steps that explained in Section 2.3., we added UT-7/Epo [17] cells by using a coculture filter (Corning Incorporate, USA). To induce hypoxia, the cells were incubated under 5% O_2_ for the last 6 hours of 24-hour period. When TNF-*α* incubation was completed to 24 hours, UT-7/Epo cells were collected and stuck on a slide with a centrifuge. The TUNEL analysis was performed by using the DeadEnd Colorimetric TUNEL System (Promega, USA). The number of apoptotic cells was analyzed under a light microscope by counting randomly 10 cells in randomly chosen 10 areas.

### 2.5. Statistical Analysis

Data were expressed as mean ± standard deviation. The Kolmogorov–Smirnov test was used to assess the normality of the distribution of the investigated parameters. Differences were tested by one-way ANOVA with Tukey's test as post hoc and Kruskal–Wallis with Dunn's test as post hoc for normally distributed or not, respectively. The values *P* < 0.05 were considered statistically significant. Statistical analysis was performed using GraphPad Prism6 statistical software (San Diego, USA).

## 3. Results

**Figure 1:**
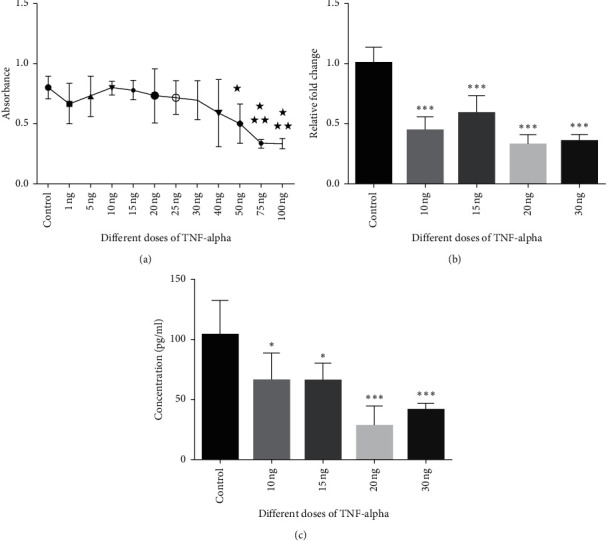
HepG2 cells were treated with different doses of TNF-*α* to detect the optimum dose. After detecting the safe doses of TNF-*α* in HepG2 cells, we incubated the cells with 4 different doses of TNF-*α*. Epo mRNA was inhibited by TNF-*α* in all doses but in 20 ng/ml dose inhibited Epo in the medium more significantly. Control: treated with normal medium. 1 ng, 5 ng, 10 ng, 15 ng, 20 ng, 25 ng, 30 ng, 40 ng, 50 ng, 75 ng, and 100 ng: treated with different doses of TNF-*α*. ^*∗*^*p* < 0.05 and ^*∗∗∗*^*p* < 0.001. (a) MTT. (b) Epo mRNA qRT-PCR analysis. (c) ELISA analysis.

In our MTT analysis, TNF-*α* seemed to be safe up to 40 ng/ml (Figure 1(a)). Regarding the previous studies, we treated the cells with 10, 15, 25, 20, and 30 ng/ml dose of TNF-*α*. According to the control group, which was not treated with TNF-*α*, all the doses inhibited the Epo synthesis in the mRNA level and protein level, whereas 20 ng/ml is the most effective dose (Figures 1(b) and 1(c)).

**Figure 2:**
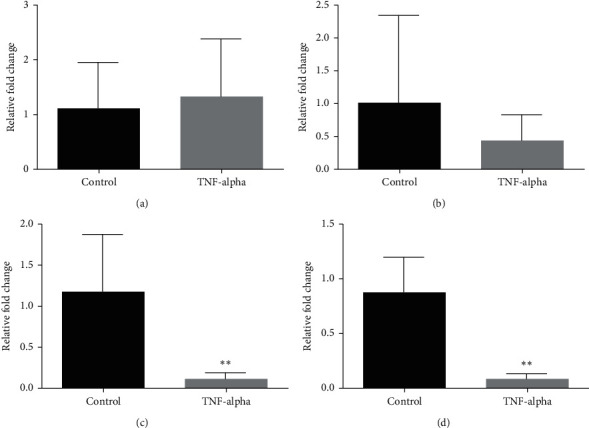
Results of qRT-PCR analysis of the miRNAs, isolated from HepG2 cells treated with 20 ng/ml TNF-*α*, that we decided to confirm from the array results. miR4459 and miR663a showed similar downregulation with array results, whereas miR122-5p and miR149-3p did not. Control: only treated with normal medium, and TNF-*α*: treated with 20 ng/ml TNF-*α*. ^*∗∗*^*p* < 0.01. (a) miR122-5p qRT-PCR analysis. (b) miR149-3p qRT-PCR analysis. (c) miR4459 qRT-PCR analysis. (d) miR663a-5p qRT-PCR analysis.

99 miRNAs showed 2 or more folded changes in our array analysis (Table 1). 8 of them had the potential to affect Epo: 1 upregulated and 7 downregulated (Table 2). 1 upregulated, miR122-5p, was not confirmed, and within the statistically most downregulated 3, miR149-3p was not confirmed, and miR4459 and miR663a were confirmed according to our confirmation study (Figure 2).

**Figure 3:**
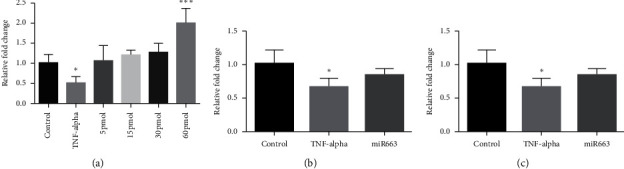
30 pmol dose of mimic-miR663a was detected as the optimum dose (a). Treatment of HepG2 cells with 30 pmol mimic-miR663a in normoxia and hypoxia prevented the inhibition of Epo synthesis against TNF-*α* (a, b). Control: treated with normal medium; TNF-*α*: treated with 20 ng/ml TNF-*α*; 5 pmol, 15 pmol, 30 pmol, 60 pmol: treated with TNF-*α* and different doses of mimic-miR663a; and miR663: treated with 20 ng/ml TNF-*α* and 5 pmol mimic-miR663a. ^*∗*^*p* < 0.05 and ^*∗∗∗*^*p* < 0.001. qRT-PCR result of (a) Epo mRNA in miR663-mimic-treated cells, (b) Epo mRNA HEPG2 cells in normoxia, and (c) Epo mRNA HEPG2 cells in hypoxia.

**Figure 4:**
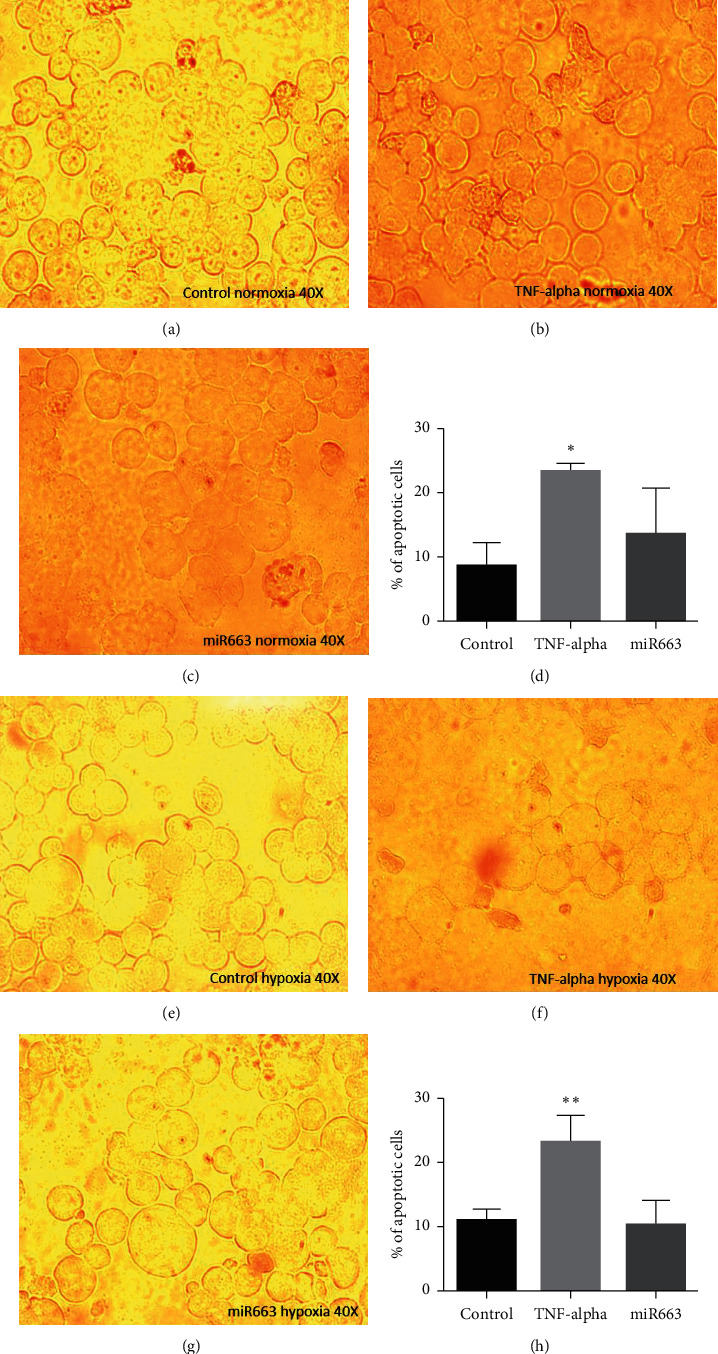
Results of the coculture study. When HepG2 cells were treated with 30 pmol mimic-miR663a, they synthesized enough Epo to let the UT-7/Epo cells survive, and when HepG2 cells were only treated with TNF-*α*, UT-7/Epo cells showed apoptosis in our coculture experiments. Both in normoxia and hypoxia showed similar results. Control: treated with normal medium; TNF-*α*: treated with 20 ng/ml TNF-*α*; and miR663: treated with 20 ng/ml TNF-*α* and 5 pmol mimic-miR663a. ^*∗*^*p* < 0.05 and ^*∗∗*^*p* < 0.01. (a) Control hypoxia 40X. (b) miR663 hypoxia 40X. (c) TNF-alpha hypoxia 40X. (d) TUNEL analysis of UT-7 Epo cells cocultured with HEPG2 in normoxia. (e) Control hypoxia 40X. (f) miR663 hypoxia 40X. (g) TNF-alpha hypoxia 40X. (h) TUNEL analysis of UT-7 Epo cells cocultured with HEPG2 in hypoxia.

After detecting the optimum dose of mimic-miR663a as 5 pmol, our qRT-PCR findings showed that mimic-miR663a prevented the Epo inhibition both in normoxia and hypoxia (Figure 3). Moreover, our coculture study confirmed these results (Figure 4).

**Figure 5:**
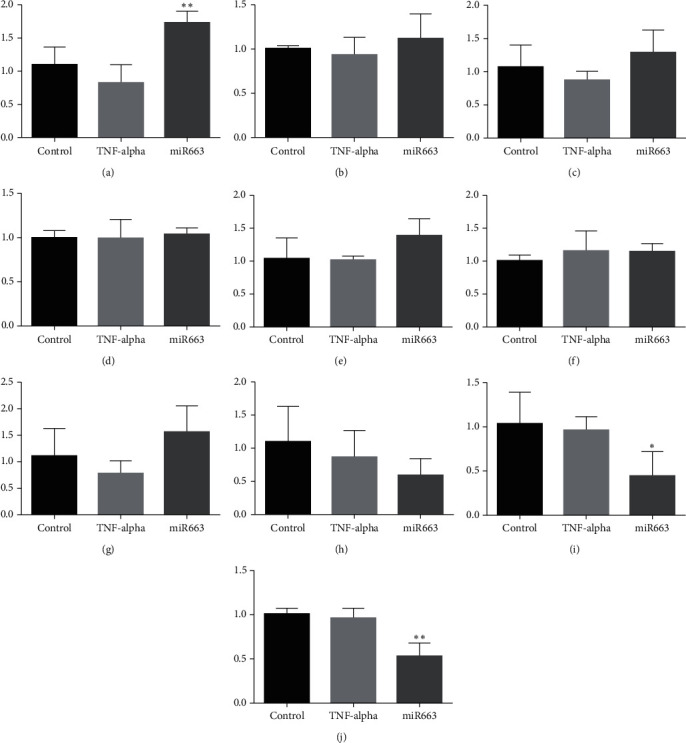
qRT-PCR results of possible mRNAs that miR663a may effect. HIF2-alpha increased significantly in normoxic conditions, and NKIRAS decreased significantly both in normoxic and hypoxic conditions. HIF1-alpha, NfKappaB, and NFKR did not show any difference. Control: treated with normal medium TNF-*α*: treated with 20 ng/ml TNF-*α*; and miR663: treated with 20 ng/ml TNF-*α* and 5 pmol mimic-miR663a. ^*∗*^*p* < 0.05 and ^*∗∗*^*p* < 0.01. PCR result of (a) HIF2-alpha mRNA in normoxia, (b) HIF2-alpha mRNA in hypoxia, (c) HIF1-alpha mRNA in normoxia, (d) HIF1-alpha mRNA in hypoxia,. (e) NfKappaB (p65) mRNA in normoxia, (f) NfKappaB (p65) mRNA in hypoxia, (g) NFKR mRNA in normoxia, and (h) NFKR mRNA in hypoxia.

Within the possible mRNAs that mimic-miR663a may also affect, HIF1-alpha, NfKappaB, and NFKR did not show any difference, whereas HIF2-alpha significantly increased and NKIRAS significantly decreased according to our qRT-PCR results (Figure 5).

## 4. Discussion

With the emergence of the role of miRNAs in the regulation of protein synthesis, it is important to examine the changes in miRNA levels at the onset and progression of diseases and also in order to formulate new treatment strategies. Today, the use of miRNAs as a drug seems to be very close [18].

The mechanism of Epo synthesis and specifically its inhibition in chronic inflammatory diseases have not yet been fully elucidated. As for miRNAs that play a role in the mechanism of Epo synthesis, we are at the very beginning of the road. In our study, we tried to detect miRNAs that are involved in the inhibition of Epo synthesis, which is inhibited in chronic inflammation, concerning similar studies.

It has been reported in previous studies that TNF-*α*, which is the main cytokine of chronic inflammatory diseases, is also the main mediator of Epo inhibition in these diseases [9]. Similar studies in HEPG2 cells, which express detectable levels of Epo even under normoxic conditions, have been reported to suppress Epo synthesis when TNF-*α* is given in the 5–10 ng/ml dose range and has been used as an in vitro chronic inflammatory experimental model [9, 10, 15]. However, we had to optimize the dose of TNF-*α* previously used for our own laboratory conditions. We showed that TNF-*α* did not have cytotoxic effects up to a dose of 40 ng/ml, utilizing the MTT test in a very wide dose range (Figure 1(a)). The qRT-PCR analysis on cells incubated at 10, 15, 20, and 30 ng/ml doses found that all doses effectively inhibited Epo mRNA synthesis (Figure 1(b)). In the ELISA analysis of the medium of the same cells, we observed that TNF-*α* at 20 and 30 ng/ml had more effective inhibition than 10 and 15 ng/ml doses (Figure 1(c)). Although the laboratory conditions are standardized, it is expected that the responses of the cells will vary slightly. The absence of a dose of 20 ng/ml in previous studies can be explained by the change in these laboratory conditions, as well as the need for a protein level measurement for dose detection. In similar studies, no data were reported that Epo was measured at the protein level for dose determination. Considering that the protein level would be essential in an in vivo model that mimics chronic inflammation, we decided to continue our experiments with the most effective low dose, that is, 20 ng/ml.

We detected 99 different 2 or more-folded human miRNAs by array analysis in total RNAs isolated from cells incubated with TNF-*α* at a dose of 20 ng/ml and isolated from nonincubated cells (Table 1). Then, we investigated the potential of these 99 miRNAs to bind to Epo mRNA and found that the decreasing 7 and increasing 1 miRNAs had this potential (Table 2). Since miRNAs can affect protein synthesis in a variety of ways, a reduction in miRNA can play as much a role as an increase, so we did not make an elimination through increase or decrease [19]. As miR122-5p is the only increasing miRNA, we have included it in the confirmation study. Among the rest, we have included miR149-3p, miR4459, and miR663a, which showed statistically higher signification compared with other miRNAs.

A study on miR122-5p showed that it is involved in Epo inhibition by TNF-*α*, so we included miR122-5p to the confirmation study [15]. Although the study of Rivkin et al. showed the effect of miR122-5p with well-planned experiments, this miRNA was not confirmed in our study (Figure 2(a)). For many reasons, such as the difference in the dose of TNF-*α* we use, such a difference may have occurred between the two studies. However, there is a need for further studies to support the effect of miR122-5p on Epo synthesis.

miR149-3p did not give a significant result and was not confirmed (Figure 2(b)). miR4459 and miR663a, the two miRNAs that we obtained the most different results from our array study, showed significant results and were confirmed (Figures 2(c)–2(d)). Interesting data for these two miRNAs are that they bind to the Epo mRNA: the 65-72 3 ′region for miR663a and the 73-79 3′ region for miR4459 (Table 3). This raises the question whether there may be a discovery of a regulatory region of Epo mRNA.

Although the array results showed a significant decrease and qRT-PCR results confirm miR4459, this miRNA is removed from the database as RNAseq experiments do not support the annotation of miR4459 as an miRNA. Finally, we decided to continue the transfection step of the experiment using miR663.

To optimize the transfection conditions, we first made a dose study for mimic-miR663.60 pmol mimics increased the mRNA of Epo even more than the control group, while all doses increased it to the control level (Figure 3(a)). Thus, we repeated the experiments with 5 pmol in normoxic and hypoxic conditions. In both conditions, mimic-miR663 increased the Epo mRNA level significantly and to the control level against TNF-*α* inhibition (Figures 3(b) and 3(c)).

Considering the complexity of protein expression mechanism, we decided to validate the increase of Epo mRNA with a coculture model. UT/7-Epo cells are Epo-dependent cells and show apoptosis when the lack of Epo in the medium. We cocultured HepG2 cells with UT/7-Epo cells and inhibited Epo synthesis from HepG2 cells with TNF-*α*. When mimic-miR663a was added to the medium of HepG2 cells, apoptosis of UT/7-Epo cells decreased significantly (Figure 4). For the first time, our method offered an alternative especially when considering the problems of Epo protein analysis.

miRNAs are known to show their effect directly degrading the target RNA. However, Vasudevan et al. reported that miRNAs may also activate RNAs, which means they may also have a regulatory role in protein expression [19]. miR663 may show its effect directly activating Epo mRNA even under TNF-*α* inhibition. However, we also checked the proteins related to Epo synthesis like HIF1, HIF2, and NfKappaB and proteins that may be a target of miR663 like NKIRAS and NFKR [10] (Figure 5). The increase in HIF2 mRNA, which is the main stimulant of Epo synthesis, when given mimic-miR663a has the biggest potential to explain the increase of Epo mRNA [3]. La Ferla et al. reported the role of NfKappaB in the synthesis of Epo [10]. The mRNA of NKIRAS, which is an inhibitor of NfKappaB, decreased significantly through mimic-miR663 incubation. However, NfKappaB did not show any difference, and this may be related to the mechanism of NfKappaB. NfKappaB is mostly found on the cytoplasm bound to IKappaB and gets away from IKappaB when activated to enter the nucleus [20]. The decrease in NKIRAS may result in a decrease in NfKappaB inhibition, which may increase Epo synthesis.

## 5. Conclusion

The increase of Epo mRNA and the decrease of apoptosis of UT/7-Epo cells together showed the preventing effect of miR663 on Epo synthesis against TNF-*α* inhibition for the first time. The complexity of the mechanism of miRNAs does not let us directly explain this preventing effect, but the increase in HIF2 and the decrease in NKIRAS mRNAs have the potential to explain. With further studies, miR663 may be a new way for the treatment of anemia seen in chronic inflammatory diseases.

## Figures and Tables

**Table 1:** Two or more fold-changed miRNAs detected by array analysis from the miRNAs isolated from 20 ng/ml TNF-*α*-treated or nontreated cells.

miRNA	Fold change	miRNA	Fold change
hsa-miR-6879-5p	3.93	hsa-miR-122-5p	−2.04
hsa-miR-3648	3.85	hsa-miR-502-3p	−2.05
hsa-miR-6126	3.72	hsa-let-7g-5p	−2.06
hsa-miR-4467	3.54	hsa-miR-103a-3p	−2.09
hsa-miR-92b-5p	3.49	hsa-miR-224-5p	−2.09
hsa-miR-4459	3.4	hsa-miR-629-5p	−2.11
hsa-miR-663a	3.12	hsa-miR-151a-5p	−2.12
hsa-miR-4758-5p	2.99	hsa-miR-107	−2.12
hsa-miR-3178	2.98	hsa-let-7d-5p	−2.13
hsa-miR-4665-5p	2.97	hsa-miR-106a-5p	−2.15
hsa-miR-1343-5p	2.94	hsa-miR-17-5p	−2.17
hsa-miR-6763-5p	2.89	hsa-miR-222-3p	−2.19
hsa-miR-6798-5p	2.88	hsa-miR-19b-3p	−2.22
hsa-miR-8072	2.75	hsa-miR-24-3p	−2.23
hsa-miR-4532	2.73	hsa-miR-20a-5p	−2.27
hsa-miR-1908-5p	2.72	hsa-miR-15b-5p	−2.29
hsa-miR-7107-5p	2.7	hsa-miR-34a-5p	−2.34
hsa-miR-4745-5p	2.67	hsa-miR-151a-3p	−2.36
hsa-miR-6821-5p	2.55	hsa-miR-182-5p	−2.43
hsa-miR-1469	2.46	hsa-miR-221-3p	−2.45
hsa-miR-4689	2.46	hsa-miR-28-5p	−2.47
hsa-miR-4530	2.45	hsa-miR-331-3p	−2.52
hsa-miR-4433-3p	2.42	hsa-miR-378a-3p	−2.53
hsa-miR-1587	2.3	hsa-miR-185-5p	−2.56
hsa-miR-149-3p	2.29	hsa-miR-181b-5p	−2.6
hsa-miR-1227-5p	2.29	hsa-miR-28-3p	−2.71
hsa-miR-3679-5p	2.28	hsa-miR-23a-3p	−2.72
hsa-miR-4668-5p	2.27	hsa-miR-27b-3p	−2.81
hsa-miR-7150	2.26	hsa-miR-1269a	−2.89
hsa-miR-4674	2.25	hsa-miR-1269b	−2.96
hsa-miR-663b	2.25	hsa-miR-26a-5p	−2.97
hsa-miR-4649-5p	2.22	hsa-miR-532-5p	−2.99
hsa-miR-6749-5p	2.21	hsa-miR-130b-3p	−3
hsa-miR-6727-5p	2.2	hsa-miR-25-3p	−3.01
hsa-miR-6085	2.18	hsa-miR-106b-5p	−3.11
hsa-miR-6765-5p	2.17	hsa-miR-181a-5p	−3.27
hsa-miR-4739	2.16	hsa-miR-16-5p	−3.44
hsa-miR-6813-5p	2.15	hsa-miR-20b-5p	−3.58
hsa-miR-4497	2.12	hsa-miR-455-5p	−3.81
hsa-miR-575	2.11	hsa-miR-18a-5p	−4.63
hsa-miR-6752-5p	2.07	hsa-let-7i-5p	−4.66
hsa-miR-6805-5p	2.05	hsa-miR-192-5p	−4.78
hsa-miR-328-5p	2.05	hsa-miR-200b-3p	−5.03
hsa-mir-5095	2.03	hsa-miR-29a-3p	−5.11
		hsa-miR-551a	−5.23
		hsa-miR-22-3p	−5.47
		hsa-miR-146a-5p	−6.38

**Table 2:** miRNAs have the potential to affect Epo synthesis according to the database search within the miRNA result of the array analysis.

Transcript ID (array design)	K avg (log2)	C avg (log2)	Fold change	*P* value	FDR *P* value
hsa-miR-4459	6.15	4.39	3.4	7.17*E − *06	0.0022
hsa-miR-663a	7.52	5.88	3.12	1.21*E − *05	0.0028
hsa-miR-1908-5p	8.16	6.71	2.72	0.0005	0.0278
hsa-miR-4433-3p	3.21	1.93	2.42	0.0206	0.333
hsa-miR-149-3p	8.17	6.97	2.29	0.000038	0.0055
hsa-miR-4739	5.71	4.6	2.16	5.54*E − *05	0.0069
hsa-miR-6805-5p	5.92	4.88	2.05	0.0034	0.0999
hsa-miR-122-5p	4.62	5.65	−2.04	0.0007	0.0307

**Table 3:** Epo mRNA binding positions of miR663a and miR4459.

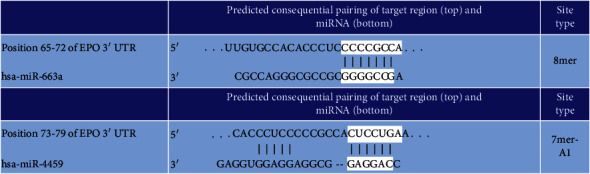

## Data Availability

The data sets used and/or analyzed during the present study are available from the corresponding author on reasonable request.
